# Pharmacokinetics of polymyxin drugs in special populations

**DOI:** 10.3389/fphar.2026.1786482

**Published:** 2026-07-03

**Authors:** Aochao Xu, Zhaoyi Tan, Liuhan Dong, Beibei Liang, Zhijian Zhang, Yun Cai

**Affiliations:** 1 The Phase I Clinical Trial Laboratory, The First Medical Center of the Chinese PLA General Hospital, Beijing, China; 2 Center of Medicine Clinical Research, Department of Pharmacy, Medical Supplies Center, Chinese PLA General Hospital, Beijing, China; 3 Department of Pulmonary and Critical Care Medicine, The Second Medical Center and National Clinical Research Center for Geriatric Diseases, Chinese PLA General Hospital, Beijing, China

**Keywords:** polymyxin B, colistin methanesulfonate, colistin sulfate, pharmacokinetics, population pharmacokinetics

## Abstract

The rising incidence of multidrug-resistant Gram-negative infections has reaffirmed the importance of polymyxins (polymyxin B (PMB), colistin methanesulfonate (CMS), and colistin sulfate (CS)) as essential last-line treatments. This review assesses the pharmacokinetic (PK) and population pharmacokinetic (PopPK) data for polymyxins in special populations, aiming to establish agent-specific PK profiles and optimize dosing strategies. A total of 72 articles published between 2005 and 2025 were reviewed, with 36 focusing on the PK of PMB, 30 on CMS, and 6 on CS. PopPK analyses constituted the majority of the included studies. The review reveals that polymyxin pharmacokinetics exhibit substantial variability across special patient groups. Key findings include an increased voxlume of distribution in critically ill patients and the identification of renal function as a major covariate influencing polymyxins PK. Altered drug clearance in patients receiving renal replacement therapies (e.g., CRRT and IHD), while body weight influences distribution in obese individuals. Distinct PK profiles were also identified in patient populations such as pediatrics, the elderly, burn victims, those on ECMO, and those with cystic fibrosis. These conditions contribute to significant variability in drug exposure, underscoring the need for model-informed precision dosing (MIPD) combined with therapeutic drug monitoring (TDM) to optimize efficacy and minimize toxicity in these vulnerable populations.

## 1 Introduction

The relentless rise of multidrug-resistant (MDR) Gram-negative bacteria, particularly the critical ESKAPE pathogens such as carbapenem-resistant *Enterobacterales*, *Pseudomonas aeruginosa*, and *Acinetobacter baumannii*, represents a global health crisis demanding urgent therapeutic solutions (Macesic et al., 2025; Sati et al., 2025). In this landscape, polymyxins such as polymyxin B (PMB), the prodrug colistimethate sodium (CMS), and colistin have re-emerged as indispensable last-line agents (Nang et al., 2021). Their pharmacokinetic-pharmacodynamic (PK/PD) characteristics are complex and clinically challenging. The ratio of the area under the concentration–time curve to the minimum inhibitory concentration (AUC/MIC) is recognized as the PK/PD index most closely associated with antibacterial efficacy. However, their clinical use is constrained by a narrow therapeutic window and a high risk of nephrotoxicity (Tsuji et al., 2019). The renewed reliance on polymyxins has driven international efforts to standardize their use. Milestones include the establishment of the first standardized clinical breakpoints by EUCAST in 2013, the WHO “Reserve” group classification in 2017, the clinical reintroduction of colistin sulfate (CS) in China in 2018, and the explicit 2022 recommendation from IDSA/SHEA for colistin as a last-resort agent (Tsuji et al., 2019; Leclercq et al., 2013; Gulland, 2017; Chinese Research Hospital Association Of Critical Care Medicine and Chinese Research Hospital Association Of Evidence Base And Translational Infectious Diseases, 2019). Despite this progress, the intrinsic complexities of polymyxin pharmacokinetics (PK) are significantly heightened in special populations. Critically ill (patients with life-threatening conditions requiring care in an intensive care unit [ICU]), renal insufficiency, elderly and pediatric patients, as well as those undergoing extracorporeal support, exhibit unique pathophysiological alterations that dramatically impact polymyxin absorption, distribution, metabolism, and excretion (Zamri et al., 2025; Zhang et al., 2025; Abdul-Aziz et al., 2020). This heightened inter-individual and intra-individual PK variability leads to unpredictable drug exposures, escalating the risk of therapeutic failure or severe toxicity, rendering current empirical dosing strategies often suboptimal for these patients (Zabidi et al., 2021; Wang X. et al., 2024; Li X. et al., 2024). This comprehensive review aims to systematically synthesize and evaluate existing PK and PopPK data for polymyxin drugs specifically within special populations to elucidate their specific PK profiles, clarify the covariates that have been shown to affect the PK of PMB, CMS, and CS.

## 2 Search strategy

Data for this review were identified by the systemic review of publications listed in the PubMed databases from the 2005 to 2025 using the following search terms (Colistin OR Polymyxins B OR Colistimethate Sodium) AND (pharmacokinetics OR PK) AND (“2005” [Date - Publication]: “2025” [Date - Publication]NOT review [Publication Type]). The starting point of 2005 was chosen because around then, studies began to show that polymyxins had acceptable efficacy and much lower toxicity than previously reported (Falagas and Kasiakou, 2005). Morover, advancements in diagnostic technologies at the start of the 21st century have enabled more accurate toxicity testing of polymyxins, revealing that their toxicity is not as severe as previously detected in the last century (Landman et al., 2008). A preliminary search identified 294 relevant articles. After screening the titles and abstracts with a specific focus on studies involving special patient populations, studies conducted in general or unselected cohorts without separate pharmacokinetic data for special populations were excluded, together with studies lacking sufficient pharmacokinetic data, *in vitro* studies, and animal experiments. Finally, 72 publications published between 2005 and 2025 were included in this review. Among them, 36 articles focused on the PK of PMB (with 21 being PopPK studies; see Table 1), 30 on CMS (with 15 being PopPK studies; see Table 2), and 6 on CS PK (with 5 being PopPK studies; see Table 3).

**TABLE 1:** A summary of published pharmacokinetic studies of PMB.

Patients Group	First author, year of publication [ref.]	Dosage	Administration interval	C_max_ (mg/L)	AUC (h·mg/L)	T_1/2_ (h)	CL (L/h)	V_d_ (L)	Model	Covariates
Healthy Subjects	Liu et al., 2021	0.75 mg/kg	Single-Dose	4.66 ± 0.461	0−24 h: 21.8 ± 1.83	5.44 ± 0.741	1.96 ± 0.14	15.33 ± 1.61	PK NCA Phoenix WinNonlin 8.0	—
					0−∞: 22.6 ± 2.04					
		1.5 mg/kg	Single-Dose	8.53 ± 1.07	0–24​h: 47.4 ± 6.39	5.55 ± 0.942	1.82 ± 0.28	14.28 ± 1.82		
					0−∞: 49.7 ± 7.08					
		0.75 mg/kg and 1.5 mg/kg	Single-Dose	—	—	—	1.89 Q_2_:9.8 Q_3_:0.448	V_1_: 4.97 ± 0.231 V_2_:4.27 ± 0.14 V_3_:3.15 ± 0.112	PopPK 3CM NONMEM 7.4	CL: Age and sex
Critical Illness	Zavascki et al., 2008	0.5–1.5 mg/kg	q12h/q48 h	2.38–13.9	20.6–61.7	—	1.134–3.402	4.97–13.58	PK 1CM PK Functions for Microsoft Excel	—
	Wang et al., 2023	N-AKI LD: 2.5 mg/kg MD: 2.5-3 mg/kg/day	q12 h	6.6 ± 2.1	0–24 h: 77.7 ± 21.8	—	—	—	PK 1CM GraphPad Prism	—
		AKI LD: 2.5 mg/kg MD: 2.5-3 mg/kg	q12 h	8.1 ± 2.2	0–24 h: 103.7 ± 31.2	—	—	—	PK 1CM GraphPad Prism	—
	Zuo et al., 2025	LD:100 MD:50	q12 h	8.3	0–24 h: 110.08	19.56	1.55	30.44	PK NCA Phoenix WinNonlin 8.1	—
	Sandri et al., 2013	0.45–3.38 mg/kg	q12 h	—	—	—	1.932 Q: 10.22	V_1_:6.573 V_2_:23.1	PopPK 2CM S-ADAPT platform	CL: TBW
​	Pi et al., 2023	75 mg (MD)	q12 h	1.61–14.53	4.1-76.01	(0.72–21.54)	1.672 Q: 7.009	V_1_: 7.857, V_2_: 12.668	PopPK 2CM Phoenix NLME	CL: Weight
										V: Weight
	Zheng et al., 2023	1.5, 2, 2.5, 3, 3.5, 4 mg/kg/day	q24 h	—	—	—	TVCL:10.6 (6.7-14.9) TVQ: 34.4 (14.7–52.2)	V_1_:33.8 (11.8–59.5) V_2_:89.2 (42.5–146.5)	PopPK 2CM NONMEM	Not included
	Liang et al., 2023	LD: 100–150 mg	q12 h	—	—	—	0.94–1.52 Q: 1.30–4.83	V_c_: 12.15–26.16 V_p_: 19.89–105.32	PopPK 2CM Pmetrics Software	CL: ALB
		MD: 50–75 mg								Age
	Wang et al., 2024b	1.5–3.0 mg/kg	q12 h	—	—	—	tvCL: 1.97 (1.80–2.14) tvQ: 3.15 (2.06–7.29)	tvV_c_: 9.84 (7.94-11.21) tvV_p_: 17.90 (10.27-24.47)	PopPK 2CM Phoenix NLME	CL: CrCL,DFI
										—
	Li et al., 2025	LD:100-150 MD:40-75	q12 h	—	—	—	CL_1_:1.39 CL_2_:13.11 CL_3_:0.186 CL_4_:3.44	V_1_:27.6 V_2_:17.1 V3:0.896 V4:4.35	PopPK 2*2CM NONMEM	CL: Age, ALB
	Yang et al., 2025	MD: 1.31 ± 0.25 mg/kg	q12 h	—	—	—	2.03	18	PopPK 1CM NONMEM	CL: CrCL, PLT
Renal Insufficiency	Kwa et al., 2011	LD:75 mg MD:50 mg	q24 h	—	B1: 16.5 Isoleucine Polymyxin B1: 4.2	B1: 11.5 Isoleucine Polymyxin B1: 18.4	B1: 3.35 Isoleucine Polymyxin B1: 1.54	B1: 55.47 Isoleucine Polymyxin B1: 40.78	PK 1CM ADAPT II program	—
	Sandri et al., 2012 CVVHD	Patient 1:75 mg; (50.8 kg)	q12 h	Patient 1:8.62	Patient 1:69.2	—	Patient 1:2.17	Patient 1: 0.50 L/kg	PK NCA Phoenix WinNonlin	—
		Patient 2:125 mg (250 kg)		Patient 2:4.38	Patient 2:75.1	​	Patient 2:6.66	Patient 2: 0.34 L/kg		
	Thamlikitkul et al., 2017	1.5–2.5 mg/kg	q12 h	—	56.0 ± 17.5	—	2.0 ± 0.6	—	PK 2CM SYSTAT	—
	Xu et al., 2022a	Normal LD: 102.89 ± 32.66 MD: 126.92 ± 14.7	q12 h	6.65 ± 1.8	0–24 h: 85.50 ± 19.5	10.2 ± 2.8	3.12 ± 0.51	36.5 ± 7.4	PK 1CM SPSS	—
		AKI LD: 124.04 ± 27.8 mg MD: 131.92 ± 15.2 mg	q12 h	6.80 ± 1.9	0–24 h: 102.37 ± 22.4	12.5 ± 3.4	2.53 ± 0.45	41.7 ± 8.3	PK 1CM SPSS	—
	Fang et al., 2024	0.75 mg/kg	Single Dose	3.4–3.8	19.6–20.4	5.71–9.07	1.88–2.32	19.2–25.6	PK NCA Phoenix WinNonlin	—
		0.75 mg/kg Long-term IHD (Intermittent Hemodialysis)		2.8 (2.5–3.1)	31.3 (30.2–32.4)	15.2 (12.8–17.6)	1.50 (1.12–1.88)	32.4 (28.1–36.7)		—
	Yu et al., 2020	2.26 mg/kg	q12 h	—	—	—	1.75 ± 0.43	20.5 ± 1.6	PopPK 1CM NONMEM	CL: CrCL
	Wang et al., 2021b	insufficiency 2 ± 0.5 mg/kg	q12 h	—	—	—	1.30-1.87	V_1_:4.78-9.18	PopPK 2CM Phoenix NLME	—
								V_2_:7.03-14.12		—
		Normal 2 ± 0.5 mg/kg	q12 h	—	—	—	1.85-2.52	V_1_: 3.62–10.13		
								V_2_: 8.56–15.38		
​	Surovoy et al., 2022b	N- CVVHD LD: 200-300 MD: 100-150	q12 h	—	—	—	2.77 ± 0.28 Q: 24.3 ± 4.3	V_1_:20.8 ± 2.24 V_2_: 90 ± 20.1	PopPK 2CM Monolix	CL: GFR
		CVVHD LD: 200-300 MD: 100-150	q12 h	—	—	—	1.87 ± 0.16 Q: 8.52 ± 5.81	V_1_:19.79 ± 5.06 V_2_: 50.56 ± 5.39		—
	Wang et al., 2022a	N-CVVHD LD: 100-200 MD: 50-100	q12 h	—	0–24 h: 77.89 ± 35.66	—	1.72–2.18 Q:0.80–3.77	V_1_: 15.0 ± 1.36 V_2_: 2.55–10.5	PopPK 2CM Phoenix NLME	CL: CrRT
		CVVHD LD: 100-200 MD: 50-100	q12 h	—	0–24 h: 27.94 ± 10.92	—	3.61–5.43 Q: 1.49–5.79	V_1_: 15.0 ± 1.36 V_2_: 12.11-20.97		—
	Luo et al., 2022	LD: 1.73 mg/kg MD: 1.66 mg/kg	q12 h	—	—	—	0.534–1.661	V_1_:10.3–13.3	PopPK 2CM NONMEM	CL: CrCL
							Q:0.898-2.444	V_2_:6.9–163 L		—
Elderly patients (≥65 years)	Zeng et al., 2024	LD: 2.0–2.5 mg/kg MD: 1.25–1.5 mg/kg	q12 h	9.23 ± 2.84	76.24 ± 41.75	11.21 ± 5.39	2.37 ± 1.76	25.50 ± 12.94	PK NCA Phoenix WinNonlin	—
	Hu et al., 2024	LD: 100 mg, MD: 75 mg	q12 h	8.31 ± 2.35	0–24 h: 117.70 ± 37.03	11.01 ± 6.28	2.10 ± 1.87	V_ss_: 22.76 ± 12.07	PK NCA Phoenix WinNonlin	—
		MD:100 mg		10.65 ± 4.60	152.73 ± 70.09	13.35 ± 5.79	1.99 ± 1.12	32.95 ± 19.51		
	Wang et al., 2022b	LD: 100–200 MD: 50–100	q12 h	—	—	—	1.59-2.14 Q:4.7-8.2	V_1_:6.94-9.33 V_2_: 14.78-24.56	PopPK 2CM Phoenix NLME	—
										V: ALB
Cystic fibrosis patients	Avedissian et al., 2018	LD: 2.98 mg/kg/day MD: 2.92 mg/kg/day	q12 h	—	—	—	7.33 Q:1.79	V_c_: 20.39 L	PopPK 2CM Pmetrics for R. Modeld	CL: CrCL
								V_2_:174.69 L		
	Crass et al., 2021	50–100 mg	q12 h	—	—	—	1.77-2.41	11.1-14.2	PopPK 1CM NONMEM	—
										V: Weight
Obesity patients	Xie et al., 2021	250 mg	q12 h	—	57.1	27.9 h	3.5 Q: 6.24	V_1_ :18.5	PK 2CM Phoenix WinNonlin	Not included
					85.7			V_2_ :82.4		
					142.9			Vss:100.9		
	Wang et al., 2021a	LD: 100–200 mg, MD: 50–100 mg	q12 h	—	—	Median: 11.24	2.37–3.36 Q:4.25–10.48	V_1_ : 8.16-14.32 V_2_ : 15.79-63.61 Vss: Median: 51	PopPK 2CM Phoenix NLME	Not included
Pediatric Patients	Xu et al., 2022b CVVHDF Patient 1 (age 3)	1:MD: 2 mg/kg	q12 h	4.94	49.4	7.1	0.5 (0.02 L/h/kg)	5.6	PK 2CM Phoenix WinNonlin	—
		MD: 2 mg/kg CVVHDF		2.6	27.1	4.2	1.3 (0.05 L/h/kg)	8.1		
	Salvatore et al., 2011 Infant (9 months)	Initial: 1.5 mg/kg	q12 h;	—	0–24 h PB1: 29.0 iso-PB1: 4.29	PB1:3.07 iso-PB1: 4.74	PB1:0.304 iso-PB1: 0.241	PB1: 1.345; iso-PB1:1.645	PK 1CM Phoenix WinNonlin	—
		Adjusted: 2 mg/kg		—	0–24 h PB1: 38.7 iso-PB1: 5.72					
	Wang et al., 2022a	1.33–2.53 mg/kg/day	q12 h	4.42 ± 1.49	36.97 ± 9.84	4.11 ± 1.41	2.27 ± 0.95	V_1_: 5.92 ± 2.10	PopPK 2CM Phoenix® NLME	CL: Weight
								V_2_: 5.53 ± 3.74		V: Weight
ECMO Patients	Surovoy et al., 2022a	100–150 mg	q12 h	5.96 ± 0.49 mg/L	48.38 ± 3.82	—	1.16 ± 0.19L/h	19.73 ± 2.02 L	PK NCA PKanalix	—
	Ye et al., 2022	LD: 2.5 mg/kg MD:1.25 mg/kg	q12 h	4.50 ± 1.35	48.76 ± 20.28	8.695 ± 1.73	1.89 ± 0.56	V_c_:11.06 ± 3.15	PopPK 2CM NONMEM	CL: CrCL
							Q: 5.18 ± 0.63	V_p_: 10.15 ± 0.84		—
Lung transplant recipients	Cai et al., 2022	LD: 2.0-2.5 mg/kg MD: 1.25-1.5 mg/kg	q12 h	—	—	—	1.72	14.4	PopPK 1CM NONMEM	CrCL
										—
Patients with liver dysfunction	Li et al., 2023	1.81 (1.54–2.31)mg/kg	q12 h	—	—	—	2.43	23.11	PopPK 1CM Phoenix NLME	CL: CrCL, Child-Pugh

Data are presented as reported in the original source literature and include mean ± standard deviation (SD), median (range), median (interquartile range), or typical population values from modeling analysis, as applicable. CRRT: continuous renal replacement therapy, CVVH: continuous veno-venous hemofiltration, CVVHD: continuous veno-venous hemodialysis, IHD: intermitted hemodialysis, ECMO: extra-corporeal membrane oxygenation, AKI: acute kidney injury, LD: loading dose, MD: maintenance dose, PB1: polymyxin B1,iso-PB: iso-polymyxin B1, C_max_: maximum plasma concentration, AUC: area under the concentration-time curve, T_1/2:_ half-life, h: hours, CL: clearance, Q Intercompartmental Clearance, V_d_: volume of distribution, V_c_/V_1_: volume of central compartment, V_p_: volume of peripheral compartment, V_2_: volume of the second compartment,V_3_: volume of the third compartment,V_4_: volume of the fourth compartment,V_ss_: volume of distribution at steady state, PK: pharmacokinetics, PopPK: population pharmacokinetics, CM: compartment model,TBW: total body weight, ALB: albumin, CrCL: creatinine clearance, DFI: drug-food interaction, PLT: platelet, GFR: glomerular filtration rate, Child-Pugh: Child-Pugh Classification.

**TABLE 2:** A summary of published pharmacokinetic studies of colistin methanesulfonate (CMS).

Patients Group	First author, year of publication [ref.]	Dosage	Administration	C_max_ (mg/L)	AUC (h·mg/L)	T_1/2_ (h)	CL (L/h)	V_d_ (L)	Model	Covariate
Healthy subjects	Mizuyachi et al., 2011	CMS: (2.5 mg/kg) 160 mg	Single dose;	CMS: 17.97 ± 3.67 Colistin: 2.55 ± 1.28	CMS: 20.82 ± 5.86 Colistin: 17.56 ± 6.80	CMS: 0.73 ± 0.30 Colistin: 4.00 ± 0.74	CMS: 20.65 ± 6.79 Colistin: 17.85 ± 5.95	CMS: 18.76 ± 3.08 Colistin: 101.01 ± 32.13	PK NCA Phoenix WinNonlin	—
		CMS 66.7 mg CBA	q12 h	CMS: 17.21 ± 2.50 Colistin: 4.38 ± 1.56	CMS: 16.11 ± 4.56 Colistin: 25.70 ± 7.49	CMS: 0.47 ± 0.17 Colistin: 4.98 ± 0.99	CMS: 26.81 ± 9.45 Colistin: 10.43 ± 3.29	CMS: 17.43 ± 3.08 Colistin: 72.24 ± 16.59	PK NCA Phoenix WinNonlin	—
	Zhao, M et al., 2018	2.4 mg CBA/kg 336 mg CMS = 150 mg CBA	Single dose	CAMS:18.0 Colistin: 0.69	CMS: 35.5 ± 4.33 Colistin: 5.87 ± 0.76	CMS: 1.38 Colistin:4.49	CMS: 9.12 Colistin: 10.68	CMS:18 Colistin: 68.2	PK NCA Phoenix WinNonlin	—
	Couet, W et al., 2011	80mgCMS (33.4mgCBA)	Single dose	CMS: 4.8 Colistin: 0.83	—	CMS:2 Colistin:3	CMS:8.88 Colistin: 2.92	CMS:14 Colistin: 12.4	PopPK 1CM NONMEM	—
Critically ill adult patients	Markou et al., 2008	CMS: 225 mg	q8h	CMS: 1.68–5.14 Colistin: 1.15–4.62	CMS:7.5–20.0 Colistin:6.0–17.2	CMS: 4.5–9.5 Colistin: 3.8–8.9	CMS: 7.8–23.0 Colistin: 9.0–26.0	CMS: 72.1–250.5 Colistin: 93.3–294.2	PK NCA Phoenix WinNonlin	—
	Imberti et al., 2010	CMS: 175 mg	q8h	Colistin: 2.21 ± 1.08	AUC_0-8_: 11.5 ± 6.2.	5.9 ± 2.6	18.2 ± 12.6	105 ± 77	PK NCA KINETICA 4.0	—
	Karnik et al., 2013)	CMS: 2 MIU or 5MIU	q8h	Colistin: 2.5-23.2 (single) Colistin: 1.8–21.8 (steady)	single 0-8: 5.4–16.4 0-inf:5.6–18.9 Steady: 0-8: 5.0–26.7 0-inf:5.2–41.8	single: 1.1–4.6 steady: 1.2–5.4	single: 1.06–2.22 steady state 2.94–7.98	14–35	PK NCA WinNonlin	—
​	Moni et al., 2020	270 mg CBA	q8h	Colistin: 2.39 ± 1.36	Colistin: 23.8 ± 11.5	—	—	CMS: 14.07 ± 10.4 Colistin: 9.9 ± 4.8	PK NCA Phoenix™WinNonlin®	—
	Plachouras et al., 2009	CMS: 3 MIU (240 mg)	q8h	—	—	CMS: 2.3 Colistin: 14.4	CMS: 13.7 Q:133	CMS: V_1_: 13.5 V_2_: 28.9; Colistin: 9.09	PopPK CMS:2CM, Colistin: 1CM NONMEM	—
	Garonzik et al., 2011	75-410 mg CBA	q12 h	—	—	—	CMS: 7.98 Colistin: 2.72	CMS: V_1_: 11.5 V_2_: 18.7 Colistin: V3:45.1	PopPK CMS:2CM Colistin: 1CM. S-ADAPT	CL: CrCL
										—
	Mohamed et al., 2012	LD: 480 mg MD: 80–240 mg	q8h	—	—	CMS: 2.2 Colistin: 18.5	CMS: 13.1 (11.6–15.1) Colistin: 8.2 (6.0–11.2) Q:206 (93–641)	CMS: V_1_: 11.8 (7.6–12.5) V_2_: 28.4 (22.4–33.5) Colistin: 218 (192–252)	PopPK CMS: 2CM Colistin: 1CM NONMEM	Not included
	Grégoire et al., 2014	Physician-Guided Dosing	q12-q8h	—	—	—	CMS:4.11 Colistin: 2.26	CMS: 15.7 Colistin: 10.2	PopPK 1CM Monolix	CL: CMS: CrCL Colistin: Plasma urea
										V: CMS: Weight Colistin: Body temperature
​	Karaiskos et al., 2015	LD: CMS (270 mg colistin) MD: CMS (140 mg colistin)	q12 h	CMS:2.65 ± 1.1 mg/L (0.95–5.1 mg/L)	—	CMS: 1.0–3.2 Colistin: 11.2 ± 3.8	CMS: 5.84 ± 2.3 Colistin: 4.99 ± 1.7	CMS V_1_: 1.42 ± 0.65 V_2_: 12.5 ± 5.3 Colistin: 80.4 ± 25.6	PopPK CMS: 4CM Colistin: 1CM NONMEM	CL: CrCL
										—
Renal Insufficiency	Markou et al., 2012	CMS: 75–150 mg	q8h-q18 h (depending on patient condition)	Colistin: Patient 1: 2.98 Patient 2: 2.29 Patient 3: 2.11	—	Patient 1: 15.7 Patient 2: 8.0 Patient 3: 7.7	Patient 1: 3.3 Patient 2: 4.3 Patient 3: 4.5	—	PK NCA Phoenix WinNonlin	—
	Karvanen M et al., 2013 CVVHDF	160 mg CMS	q8h	CMS: 6.92 ± 2.83 Colistin: 0.92 ± 0.46	—	CMS: 3.3 h	CMS: 8.23 ± 3.07 Colistin: 18.91 ± 5.95	—	PK 2CM Prisma	—
	Menna et al., 2018	Patient 1: LD: 720 mg MD: 240 mg	q8h	Colistin: 13.4	216.3	—	Patient 1: 4.2	64	PK NCA Prisma	—
		Patient 2: LD: 720 mg MD: 160 mg	q12 h	Colistin: 10.9	179.1	—	Patient 2: 5.0	90		
	Schmidt et al., 2019	LD: 6MIU CMS MD: 3MIU CMS	q8h	CMS: 8.12–12.32. Colistin: 8.39–10.41	CMS: 58.2–73.2 Colistin: 106.9–189.2	—	CMS: 3.378–19.122 Colistin: 2.196–5.772	—	PK NCA GraphPad Prism.	—
	Koomanachai et al., 2014	MD: 150mgCBA	q24 h	—	—	CMS:8.4 Colistin:13.2	CMS: 1.77 Colistin: 2.74	CMS: V_1_: 11 Colistin: V_2_:54.2	PopPK CMS:2CM Colistin:1CM S-ADAPT (version 1.57)	CL: Weight
										—
​	Jitmuang et al., 2015 haemodialysis	150 mg CMS.	q24 h	—	—	CMS: 11.9 Colistin:24.8	CMS: HD: 4.26–5.67 N-HD: 2.66 Colistin: 3.99	CMS: V_1_:11.8 V_2_:11.8 Colistin: 141	PopPK CMS:2CM Colistin:1CM S-ADAPT	Not included
	Karaiskos et al., 2016	LD: 270 mg CBA MD: 140 mg CBA	q12 h	CMS:6.5–29.9 Colistin: 0.5–3.02	—	CMS:/. Colistin: 9.2–18.5	CMS: 0.95–1.35 Colistin: 2.45–3.55	—	PopPK 1CM NONMEM	—
	Leuppi-Taegtmeyer et al., 2019 CRRT	LD: 9 MIU (6 MIU for <60 kg), MD: 3 MIU (2 MIU for <60 kg).	q8h	—	—	—	CMS: 2.31 Colistin: 1.93	CMS: 12.1 Colistin: 70.1	PopPK 6CM NONMEM	CL: Ht
	Boonyasiri et al., 2025	LD:300 mg CBA MD:150 mg CBA	q12 h or q24 h	​	​	​	1.69	50.2	PopPK 1CM NONMEM	—
Burn patients	Healy et al., 2011 17 years boy	2.5 mg/kg CMS	q24 h	Colistin: 3.6 ± 1.0 (peak)	47.1 ± 14.4	12.3 ± 9.4 (off dialysis) 9.4 ± 4.6 (on dialysis)	0.312 ± 0.102 (5.2 ± 1.7 mL/min)	—	PK NCA Not included	—
	Akers et al., 2015 CVVH	75 mg CMS	q12 h	Colistin: Patient1: 13.6	1:222.2	1:22	2.7	86.1	PK NCA Phoenix WinNonLin	—
			q8h	Colistin: Patient2: 6.7	2: 100.0	2:11.9	4.7	99.7		
	Corcione et al., 2017	1.5–2MIU CMS	q12 h	4.05-18.82	16.84-162.81	3.51-10.43	2.25-14.21	18.58-25.66	PK NCA Kinetica 5.0	—
	Lee et al., 2013	150 mg CBA	q12 h	—	Colistin: 29.2 ± 13.5	Colistin: 6.6	Colistin: 8.49–10.8	Colistin: 59.6–113.5	PopPK 1CM NONMEM	CL: CrCL
										—
Pediatric patient	Nakwan et al., 2016	5 mg/kg CBA	Single dose	Colistin: 3.0 ± 0.7	Colistin: 25.3 ± 10.4	Colistin: 9.0 ± 6.5	Colistin: 0.6 ± 0.3 L/h/kg	Colistin: 7.7 ± 9.3 L/kg	PK NCA KINETICA 4.0	—
	Wacharachaisurapol et al., 2020	LD: 4 mg of CBA//kg MD: 1.7–2.5 mg of CBA/kg	q12 h (2.5 mg)	CMS: 6.3 ± 3.0	CMS: 29.0 ± 16.5	CMS: 3.3 ± 0.4	CMS: 0.2 ± 0.1 L/h/kg	CMS: 0.8 ± 0.5 L/kg	PK NCA PK Solver program	—
			q8h (1.7 mg)	CMS: 6.0 ± 1.6	CMS: 24.5 ± 7.3	CMS: 2.5 ± 0.6	CMS: 0.2 ± 0.0 L/h/kg	CMS: 0.6 ± 0.2 L/kg		
		MD: 1.7–2.5 mg of CBA/kg	q12 h (2.5 mg)	CMS: 3.7 ± 1.1	CMS: 12.2 ± 2.9	CMS: 2.7 ± 0.5	CMS: 0.2 ± 0.0 L/h/kg	CMS: 0.8 ± 0.2 L/kg		
			q8h (1.7 mg)	CMS: 4.5 ± 1.4	CMS: 14.8 ± 3.7	CMS: 2.6 ± 0.3	CMS: 0.1 ± 0.0 L/h/kg	CMS: 0.4 ± 0.1 L/kg		
	Magreault et al., 2018	3 MIU (90 mg CBA)	q8h	CMS: 12.9 Colistin: 0.5–1.5	—	—	CMS: 12.3 Colistin: 19.92	CMS: 10.1	PK 1CM Monolix Suite 2016R1	—
	Ooi et al., 2019	6.6 mg CBA/kg/day	q8h	Colistin: Css,avg: 0.41–3.50	—	CMS: 1.29–3.73 Colistin: 2.28–6.07	CMS: 0.213–3.51 Colistin: 0.274–2.82	CMS: 0.824–4.31 Colistin: 2.22–14.7	PopPK CMS:2CM Colistin:1CM S-ADAPT	CL: CrCL
										—
	Wacharachaisurapol et al., 2021	5 mg of CBA/kg/day	q12 h	—	—	—	Colistin:4.83	Colistin: 46.06	PopPK Non-linear mixed-effects mode Phoenix version	CL: Scr
										—
	Antachopoulos et al., 2021	200,000–350,000 IU/kg/day (6.6–11.6 mg CBA/kg/day)	q12 h or q8h	—	—	Colistin:4.96	Colistin:4.1	Colistin: 20.7	PopPK 1CM NONMEM	CL: CrCL, SIRS
										V: Weight
Cystic fibrosis patients	Yapa et al., 2014	150 mg CBA; Inhaled: 2 MIU and 4 MIU CMS	q8h	Colistin: 0.40–0.77	CMS: 46.9–75.1 Colistin: 1.39–4.10	CMS: 2.66 ± 0.66 Colistin: 7.34 ± 1.41	CMS: 5.96 ± 1.07	CMS: 16.9 ± 4.68	PK NCA Phoenix WinNonlin	—
ECMO Patients	Suk et al., 2023, Suk et al., 2025	LD:9MIU or 12MIU MD:4.5MIU	q12 h or q8h	—	—	—	CMS:1.83 Colistin:0.32	CMS:10.96	PopPK 1CM Monolix	CL: CrCL
										—

Data are presented as reported in the original source literature and include mean ± standard deviation (SD), median (range), median (interquartile range), or typical population values from modeling analysis, as applicable.

ECMO: extra-corporeal membrane oxygenation, CRRT: continuous renal replacement therapy, CVVHDF: continuous venovenous hemodiafiltration, CBA: colistin base activity, LD: loading dose, MD: maintenance dose, C_max_: maximum plasma concentration, AUC: area under the concentration-time curve, T_1_/_2_: half-life, h: hours, CL: clearance, Q: intercompartmental clearance, V_d_: volume of distribution, V_c_/V_1_: volume of central compartment, V_p_: volume of peripheral compartment, V_2_: volume of the second compartment, PK: pharmacokinetics, PopPK: population pharmacokinetics, CM: compartment.

CrCL: creatinine clearance, Scr: serum creatinineb, Ht: hematocrit, SIRS: systemic inflammatory response syndrome.

**TABLE 3:** A summary of published pharmacokinetic studies of Colistin Sulfate.

Patients Group	First author, year of publication [ref.]	Dosage and administration	Administration	C_max_ (mg/L)	AUC (h·mg/L)	T_1/2_ (h)	CL (L/h)	V_d_ (L)	Model	Covariate
Healthy subjects	Huang et al., 2024	Single dose: 0.452 mg/kg	Single dose:	1.08 ± 0.18	4.73 ± 0.89	3.65 ± 0.55	3.24 ± 0.51	16.82 ± 2.70	PK NCA Phoenix WinNonlin	—
		MD: 0.452 mg/kg	q12 h	1.10 ± 0.15	5.33 ± 0.71	4.53 ± 1.22	3.26 ± 0.48	17.32 ± 2.52		—
Critically ill patients	Yu et al., 2022	0.5 MIU 0.75 MIU 1 MIU	q12 h	0.28–6.20	39.39 ± 14.47	5.3 ± 1.2	1.74 ± 0.61	20.7	PopPK 1CM NONMEM	CL: CrCL
										—
	Xie et al., 2022	1.0 MIU, 1.5 MIU, 2.0 MIU	q12 h	—	—	—	1.50 (1.15–1.84) Q:1.71 (0.961–2.45)	V_1_:16.1 (13.3–18.9) V_2_: 28.4–72.6	PopPK 2CM NONMEM	CL: CrCL
										V: ALT
	Sun et al., 2025	1.0–2.0MIU	q12 or q8h	—	—	—	2.66 Q:1.63	V_1_:49.7 V_2_:109 (58.18–161.31)	PopPK 2CM NONMEM	CL: CrCL
										V: Weight
Renal Insufficiency	Huang et al., 2025 CRRT	1.0–1.5MIU	q12 or q8h	—	—	—	3.69 Q:25.30	V_1_:20.50 V_2_:33.20 (24.44-50.87)	PopPK 2CM NONMEM	CL: CysC Weight
										—
Lung transplant patients	Cai et al., 2024	0.5 MIU, 0.75 MIU, 1.0 MIU	q12-q8h	C:0.05-4.18	0–24 h: 20.95 ± 10.13	—	2.45	15.9	PopPK 1CM NONMEM	CL: CrCL Concomitant use of furosemide
										—

MD: maintenance dose, C_max_: maximum plasma concentration, AUC: area under the concentration-time curve, T_1_/_2_: half-life, h: hours, CL: clearance, Q: intercompartmental clearance, V_d_: volume of distribution, V_1_: volume of central compartment, V_2_: volume of the second compartment, PK: pharmacokinetics, PopPK: population pharmacokinetics, CM: compartment, CrCL: creatinine clearance, Scr: serum creatini

## 3 Results

The PK profiles of PMB, CMS, and CS demonstrate significant inter-drug variability across both healthy and special populations, driven by differences in chemical structure, administration forms, and metabolic pathways. The PK parameters of polymyxin drugs in special population plasma at a steady-state after polymyxin drugs administration are summarized in Tables 1–3. Detailed information on patient demographics, study design, and external validation for these studies is provided in Tables 4–6.

**TABLE 4:** Demographics of published PK studies of PMB.

Patients group	First author, year of publication [ref.]	Number of subjects	Nationality	Age range	Study design	PopPK external validation
Healthy Subjects	Liu et al., 2021	20	China	19–45	Single-center	No
Critical Illness	Zavascki et al., 2008	8	Brazil	42–86	Single-center	No
	Wang et al., 2023	55	China	61.0 ± 16.7	Single-center	No
	Zuo et al., 2025	27	China	68.0 (31–94)	Single-center	No
	Sandri et al., 2013	24	Brazil	21–87	Single-center	No
	Pi et al., 2023	30	China	55 (21–82)	Single-center	No
	Zheng et al., 2023	9	Brazil	59.4 (18.33)	Single-center	No
	Liang et al., 2023	22	China	68 (31–94)	Single-center	No
	Wang et al., 2024a	83	China	52.0 (18.0–94.0)	Single-center	No
	Li et al., 2025	76	China	66.0 (55.0–76.3)	Single-center	No
	Yang et al., 2025	95	China	60 (47–74)	Multi-center	No
Renal Insufficiency	Wang et al., 2021b	58	China	47.3 ± 17.7 54.2 ± 17.5	Single-center	No
	Thamlikitkul et al., 2017	19	America	61.0 ± 9.4	Multi-center	No
	Xu et al., 2022a	52	China	64.7 ± 16.5	Multi-center	No
	Fang et al., 2024	22	China	44 (35, 67)	Single-center	No
	Yu et al., 2020	32	China	63.63 (12.92)	Single-center	No
	Surovoy et al., 2022a	37	Russia	44–87	Single-center	No
	Wang et al., 2022c	53	China	53.53 ± 17.95	Single-center	No
	Luo et al., 2022	63	China	65 (27–93) 61 (32–104)	Single-center	No
Elderly patients (≥65 years)	Zeng et al., 2024	23	China	70.2 ± 13.7	Single-center	No
	Hu et al., 2024	14	China	79.8 ± 8.2	Single-center	No
	Wang et al., 2022b	23	China	73.0 (65.0–94.0)	Single-center	No
Cystic fibrosis patients	Avedissian et al., 2018	9	America	32 (25-44.4)	Single-center	No
	Crass et al., 2021	9	America	21–57	Single-center	No
	Wang et al., 2021a	26	China	52 (18–83)	Single-center	No
	Wang et al., 2022a	19	China	12.5 (3.2-17.8)	Single-center	No
ECMO Patients	Surovoy et al., 2022a	13	Russia	44 ± 3.6	Single-center	No
	Ye et al., 2022	8	China	58.9 ± 9.4	Single-center	No
Lung transplant recipients	Cai et al., 2022	34	China	56 ± 12.76	Single-center	No
Patients with liver dysfunction	Li et al., 2023	138	China	58.0 (49.0–72.3)	Single-center	No

**TABLE 5:** Demographics of published PK studies of CMS.

Patients group	First author, year of publication [ref.]	Number of subjects	Nationality	Age range	Study design	PopPK external validation
Healthy subjects	Mizuyachi et al., 2011	20	China	19-45	Single-center	No
	Zhao, M et al., 2018	24	China	19-45	Single-center	No
	Couet, W et al., 2011	12	France	29.5 ± 5.5	Single-center	No
Critically ill adult patients	Markou et al., 2008	14	Greece	18-80	Single-center	No
	Imberti et al., 2010	13	Italy	20-70	Single-center	No
	Karnik et al., 2013	15	India	15-57	Single-center	No
	Moni et al., 2020	20	India	28-78	Single-center	No
	Plachouras et al., 2009	18	Greece	40-83	Single-center	No
	Garonzik et al., 2011	105	America, Thailand	19-92	Multi-center	No
	Mohamed et al., 2012	10	Greece	32-88	Single-center	No
	Grégoire et al., 2014	71	France	18–91	Multi-center	No
	Karaiskos et al., 2015	19	Greece	18-86	Single-center	No
Renal Insufficiency	Markou et al., 2012	3	Greece	45,58,62	Single-center	No
	Karvanen M et al., 2013 CVVHDF	5	Greece	57-83	Single-center	No
	Menna et al., 2018	2	Italy	58,72	Single-center	No
	Schmidt et al., 2019	8	Germany	35-63	Single-center	No
	Koomanachai et al., 2014	8	Thailand	54-77	Single-center	No
	Jitmuang et al., 2015	10	Thailand	38-66	Single-center	No
	Karaiskos et al., 2016	8	Greece	28-78	Single-center	No
	Leuppi-Taegtmeyer et al., 2019 CRRT	11	America	27-70	Single-center	No
	Boonyasiri et al., 2025	13	Thailand	33-76	Single-center	No
Burn patients	Healy et al., 2011	1	Mexican	17	Single-center	No
	Akers et al., 2015 CVVH	2	America	37	Single-center	No
			America	68	Single-center	No
	Corcione et al., 2017	8	America	31-85	Single-center	No
	(Lee et al., 2013)	50	China	26-80	Single-center	No
Pediatric patient	Nakwan et al., 2016	7	Thailand	0.46–0.77	Single-center	No
	Wacharachaisurapol et al., 2020	20	Thailand	2-18	Single-center	No
	Magreault et al., 2018	1	France	8	Single-center	No
	Ooi et al., 2019	5	America	01.-6.25	Single-center	No
	Wacharachaisurapol et al., 2021	79	Thailand	2.6 (0.8–6.8)	Multi-center	No
	Antachopoulos et al., 2021	17	America	0.3-13.75	Single-center	No
Cystic fibrosis patients	Yapa et al., 2014	6	Australia	20-35	Single-center	No
ECMO Patients	Suk et al., 2023	18	Canada	37–69	Single-center	No
	Suk et al., 2025	​	​	​	​	​

**TABLE 6:** Demographics of published PK studies of Colistin Sulfate.

Patients group	First author, year of publication [ref.]	Number of subjects	Nationality	Age range	Study design	PopPK external validation
Healthy subjects	Huang et al., 2024	Single dose: 12	China	20-38	Single-center	No
		MD: 12	China	22-43	Single-center	No
Critically ill patients	Yu et al., 2022	42	China	67.90 ± 13.74	Single-center	No
	Xie et al., 2022	20	China	18-92 (60.5)	Single-center	No
	Sun et al., 2025	178	China	31-98 (78.5)	Single-center	No
Renal Insufficiency (CRRT)	Huang et al., 2025	20	China	50.5 ± 14.1	Single-center	No
Lung transplant patients	Cai et al., 2024	39	China	55.59 (24-91)	Single-center	No

### 3.1 Healthy population: baseline PK characteristics

Studies in healthy subjects have established the foundational PK profiles for PMB, CMS/colistin, and CS.

The clearance (CL) of PMB is primarily mediated through non-renal pathways. In healthy subjects, a key finding was that when the administered dose was doubled (from 0.75 to 1.5 mg/kg), key parameters like CL and V_d_ (volume of distribution), remained unchanged, while the peak concentration increased with the dose. The average elimination half-life is approximately 5.5 h (see Table 1 for full details). PopPK studies have shown that the three-chamber model is more suitable for healthy subjects, with age and gender as covariates (Liu et al., 2021).

In contrast, CMS is metabolized via the renal pathway. As a prodrug, CMS is converted *in vivo* to its active form, colistin, which exhibits a complex PK profile. A key observation is the significant drug accumulation that occurs under multiple-dose regimens. For instance, one study demonstrated that at steady state, the C_max_ of colistin increased to 4.38 mg/L from 2.55 mg/L observed after a single dose (Mizuyachi et al., 2011). Furthermore, marked discrepancies in colistin’s PK parameters are evident across different studies. This appears to be linked to the use of different CMS formulations, which result in different administered doses of colistin base activity (CBA). For example, a study in healthy French subjects that administered a single 80 mg dose of CMS (equivalent to 33.4 mg CBA) reported a colistin V_d_​ of 12.4 L (Couet et al., 2011). In contrast, a study in healthy Chinese subjects that administered a dose equivalent to 150 mg CBA reported a much higher V_d_ of 68.2 L (Zhao et al., 2018). These data underscore that direct cross-study comparisons of PK parameters must be approached with extreme caution, given that product potency and actual active content may vary (Mizuyachi et al., 2011; Couet et al., 2011; Zhao et al., 2018).

In healthy subjects receiving CS, drug exposure did not increase over the course of a multiple-dose regimen (q12 h for 7 days). Specifically, key exposure metrics after the initial dose, such as C_max_ (1.08 mg/L) and AUC (4.73 h mg/L), were comparable to those measured at steady state (C_max_1.10 mg/L; AUC_0–12h_:5.33 h mg/L). The t_1/2_ was reported to be approximately 4 h. Furthermore, the cumulative urinary recovery of colistin was negligible, reaching only 0.9% ± 0.7% within the 24-h period after the last dose in the multi-dose group (Huang et al., 2024).

### 3.2 Critical illness

In critically ill adult patients, the PK of polymyxins is characterized by substantial inter-individual variability.

Conventional PK studies of PMB have shown significant inter-study variability, with wide and often inconsistent ranges reported for key PK parameters (Zavascki et al., 2008; Wang et al., 2023). One study reported higher mean C_max_ and AUC_0–24h_ values in critical patients with acute kidney injury (AKI) compared to those without (Wang et al., 2023).

These models identified several significant covariates impacting key PK parameters. CL was influenced by body weight (Pi et al., 2023), renal function (measured as CrCL), the administration of renal replacement therapy, albumin (ALB), and daily fluid intake (DFI) (Wang P. et al., 2024; Liang et al., 2023). The V_d_ was affected by both body weight and age (Pi et al., 2023; Liang et al., 2023). The typical values for CL and V_d_ reported in these models varied across studies, reflecting differences in critically ill adult patients and modeling methodologies.

In critically ill adult patients, the PK of CMS and its active form, colistin, are characterized by substantial inter-individual variability. PopPK studies of CMS often utilizing a two-compartment model for CMS and a one-compartment model for colistin. These analyses have identified several significant covariates. Renal function, measured by creatinine clearance (CrCL), was consistently found to influence the CL of CMS (Garonzik et al., 2011; Karaiskos et al., 2015). Other significant determinants of Vd included body weight and body temperature, with the former positively and the latter negatively correlated with Vd, hemoglobin level was statistically significantly associated with the intercompartmental clearance (Q) of CMS (Plachouras et al., 2009; Grégoire et al., 2014). Some PopPK studies analyzed the data of CS using one-compartment model or two-compartment model, respectively. A core finding of these analyses was the impact of renal function, CrCL was identified as a key covariate affecting drug CL across both models (Yu et al., 2022; Xie et al., 2022; Sun et al., 2025). Additionally, an analysis using the two-compartment model found that alanine aminotransferase (ALT) was a covariate influencing the V_d_ (Xie et al., 2022).

### 3.3 Renal impairment

The PK of polymyxins in patients with renal insufficiency are significantly affected by the level of renal function.

Clinical PK Study have shown that the PK parameters of PMB differ significantly among patients with renal impairment undergoing different renal treatment modalities. PopPK models have been developed to further quantify these effects. These analyses have consistently identified measures of renal function, such as CrCL, glomerular filtration rate (GFR), and serum creatinine (Scr), as significant covariates influencing PMB CL (Wang et al., 2022a). Furthermore, the administration of continuous renal replacement therapy (CRRT) has also been identified as a key factor affecting PMB disposition in this population (Wang et al., 2022a).

As a drug eliminated via the kidneys, CMS is more significantly affected by different renal replacement therapies (RRT) (Jitmuang et al., 2015; Leuppi-Taegtmeyer et al., 2019). PK studies focus on investigating the changes in PK parameters of CMS at different doses in this patient population undergoing different RRT (Leuppi-Taegtmeyer et al., 2019; Menna et al., 2018; Schmidt et al., 2019).

Unlike other previous studies, the latest PopPK study of CS in patients receiving CRRT has found that cystatin C (CysC) may have a more significant impact on CL than CrCL (Huang et al., 2025). This is likely because CysC is a more reliable indicator of GFR (Björk et al., 2018).

### 3.4 Elderly population

Only PMB has been specifically studied for its PK in the elderly population. In a prospective comparative study, found that while the AUC_0–24h_ of PMB was comparable between elderly and younger critically ill patients, the elderly cohort exhibited a significantly prolonged drug half-life (Zeng et al., 2024). Another PK study assess two fixed-dose PMB regimens and determined that both 75 mg and 100 mg maintenance doses resulted in excessive drug exposure, suggesting these doses may be too high for the elderly population (Hu et al., 2024). PopPK analysis of elderly patients described PMB disposition using a two-compartment model and identified serum albumin as a significant covariate for the V_d_ (Wang et al., 2022b).

### 3.5 Pediatric populations

PK data for PMB in this population are primarily derived from case reports and a single PopPK study. PopPK study have shown that both CL and V_d_ of PMB increase with weight (Wang P.-L. et al., 2022). In contrast, PK data for CMS, have been generated through more PK studies. Non-compartmental analysis (NCA) studies of CMS have provided PK parameters for pediatric groups. A study investigated CMS PK in children under various multiple-dose regimens, giving a CMS loading dose of 4 mg of CBA/kg is beneficial for the improvement of drug exposure by the increasing of AUC and C_max_ (Wacharachaisurapol et al., 2020). PopPK modeling has been employed to further describe the disposition of CMS and colistin in children and to identify sources of variability. These studies have identified renal function, measured by CrCL or Scr, as a significant covariate influencing colistin CL (Ooi et al., 2019; Wacharachaisurapol et al., 2021; Antachopoulos et al., 2021). One analysis further reported that the presence of Systemic Inflammatory Response Syndrome (SIRS) and body weight impacted CMS PK parameters (Antachopoulos et al., 2021).

### 3.6 Cystic fibrosis (CF)

PopPK studies have evaluated PMB in patients with CF, applying both one- and two-compartment models. Separate analyses have identified CrCL as a significant covariate on CL, while body weight has been identified as a covariate for the V_d_ (Avedissian et al., 2018; Crass et al., 2021).

The PK of CMS and its conversion to colistin have been described in CF patients following both intravenous (IV) and inhaled administration. A NCA of IV CMS characterized its CL (5.96 ± 1.07 L/h) and V_d_ (16.9 ± 4.68 L), while also evaluating the systemic colistin exposure (C_max_: 0.53 ± 0.11 mg/L and AUC: 7.24 ± 2.01 mg h/L) resulting from single IV infusion of CMS (Yapa et al., 2014).

### 3.7 Lung transplant recipients

A one-compartment PopPK model has been used to describe PMB disposition in lung transplant recipients. The analysis identified CrCL as a significant covariate on PMB CL (Cai et al., 2022). The PK of colistin in lung transplant patients have also been characterized using a one-compartment PopPK model. This analysis identified renal function and concomitant furosemide use as significant covariates (Cai et al., 2024).

### 3.8 Extracorporeal membrane oxygenation (ECMO) patients

In patients requiring ECMO support, PK studies for both PMB and CMS indicate the extracorporeal circuit does not significantly impact drug disposition. Renal function remains the primary determinant of CL, as a two-compartment PopPK model for PMB and a one-compartment PopPK study for CMS both identified CrCL as a significant covariate (Suk et al., 2025; Ye et al., 2022). Consequently, current evidence suggests that dose adjustment is not necessary for patients receiving ECMO alone, as ECMO circuits exhibit low drug adsorption and do not primarily function for polymyxins clearance (Suk et al., 2025; Ye et al., 2022; Surovoy et al., 2022a).

### 3.9 Obesity patients

For obese patients, PK studies are available only for PMB, with a primary focus on informing dosing strategies.

The PopPK study suggest that adjusted body weight (ABW)-based regimens with a daily dose not exceeding 250 mg have a high likelihood of achieving an AUC_ss,24h_ of 50–100 mg h/L and attaining PK/PD targets for pathogens with an MIC ≤0.5 mg/L (Wang et al., 2021a). Both standard and PopPK analyses have been conducted using two-compartment models. These studies provided estimates for key parameters, though neither identified significant covariates influencing its disposition (Wang et al., 2021a; Xie et al., 2021).

### 3.10 Burn patients

PK data in burn patients are currently available only for CMS. A one-compartment PopPK analysis has characterized colistin disposition in this population, identifying CrCL as a significant covariate on its CL (Lee et al., 2013). Additionally, a PK study of colistin CMS in burn patients find that total body surface area (TBSA) affected PK parameters (Corcione et al., 2017). This association may be explained by burn-related pathophysiological changes, as larger TBSA burns are often associated with more extensive capillary leakage, fluid resuscitation, hypoalbuminemia, expanded extracellular fluid volume, and altered renal blood flow, all of which may influence drug distribution and clearance (Lee et al., 2013; Corcione et al., 2017; Healy et al., 2011; Akers et al., 2015).

## 4 Discussion

This review systematically consolidates the current understanding of the PK of the polymyxins (PMB, CMS and CS), across a range of special patient populations. The findings demonstrate that, unlike the relatively predictable PK profiles observed in healthy volunteers, the pathophysiological states of these special populations lead to significant inter-individual and intra-individual PK variability. The majority of polymyxins studies utilized a two-compartment model or one-compartment model adequately, while a significant minority found more complex model. This divergence is likely a consequence of varying sampling strategies rather than biology itself. Studies with sparse sampling may fail to capture the distribution phase, simplifying the model and potentially mischaracterizing the drug’s profile (Chen et al., 2022).

In healthy subjects, the intrinsic metabolism of these drugs is best defined: CMS is primarily cleared renally unchanged, while CS and PMB undergo complex, non-renal dominant elimination. This baseline is crucial for understanding alterations in special populations (Liu et al., 2021; Zhao et al., 2018; Huang et al., 2024). Overall, renal function was the most repeatedly identified and clinically actionable covariate across polymyxin pharmacokinetic studies, particularly for CMS/colistin and CS, whereas its role in PMB disposition was less consistent. For CMS: Its CL is unequivocally and predictably correlated with CrCL, renal function determines not only the elimination of CMS itself but also the extent of systemic colistin formation. In patients with preserved renal function, a substantial fraction of CMS may be excreted unchanged before conversion, whereas renal impairment prolongs CMS residence time, thereby increasing the opportunity for *in vivo* conversion to colistin and increasing colistin exposure, making renal function the primary driver for dose adjustment (Zabidi et al., 2021; Kristoffersson et al., 2020). In contrast, the situation for PMB and CS is more complex and remains debated, PMB is administered as an active compound and undergoes predominantly non-renal elimination, with only a small fraction recovered unchanged in urine. Its disposition may involve extensive tissue binding, uptake by renal and non-renal tissues, and intracellular degradation, rather than simple glomerular filtration (Avedissian et al., 2019). The PopPK studies investigating PMB, only eight concluded CrCL was a statistically significant covariate for CL. This controversy likely arises from the non-renal metabolic mechanisms of PMB, the mixed and inconclusive evidence regarding the link between its CL and renal function suggests that renal function-based dosing adjustment may not be necessary (Wang et al., 2021b). In terms of CS, a recent study have shown, CysC is a significant variable influencing CL and outperforms CrCL in this regard (Huang et al., 2025). Special populations induce profound PK alterations, but the specifics differ between CMS, CS and PMB. Critically illness is the most challenging population. Systemic inflammatory response and capillary leak syndrome can significantly increase the V_d_ of polymyxins (Zavascki et al., 2008; Zuo et al., 2025; Li L. et al., 2024). Critically ill and elderly patients commonly present with hypoalbuminemia, which reduces the protein binding rate of PMB and increases the concentration of free drugs, may be one of the reasons for the increased toxicity risk of PMB in this population (Liang et al., 2023; Nation et al., 2019). As a nephrotoxic agent, polymyxins require particularly cautious use in patients with renal impairment. Renal impairment not only affects the CL of CMS and colistin but also influences the conversion of CMS to its active form, colistin (Leuppi-Taegtmeyer et al., 2019; Fiaccad et al., 2016). CRRT modalities such as continuous veno-venous hemodiafiltration (CVVHDF) and continuous veno-venous hemodialysis (CVVHD) affect the CL of CMS (Wang et al., 2022a; Leuppi-Taegtmeyer et al., 2019). Even a PopPK study found that hematocrit (Ht) as a covariate affecting the efficacy of blood transfer (Q_blood_) in and out of the filter improved the model significantly (Leuppi-Taegtmeyer et al., 2019). Alongside these findings, continuous venovenous haemofiltration (CVVH) has also been shown to significantly increased the CL of PMB (Wang et al., 2022a). ECMO, another form of extracorporeal therapy, however, does not affect the PK of either CMS or PMB. Instead, CrCL remains the significant influencing factor (Suk et al., 2025; Ye et al., 2022; Surovoy et al., 2022a). This limited effect is attributable to low drug adsorption and non-clearance-dominant nature of ECMO circuits (Kim et al., 2024). As suggested in the literature, routine dose adjustment of polymyxins is unnecessary for ECMO patients (Suk et al., 2025; Ye et al., 2022; Kim et al., 2024).

Certain scenarios may apply exclusively to specific populations. For instance, the Child-Pugh score significantly influences the CL of PMB in patients with hepatic impairment; the total body surface area affects the CL of CMS in burn patients and in lung transplant recipients receiving CS, whether furosemide is co-administered or not has also been found to exert a significant impact on the CL of CS (Cai et al., 2024; Corcione et al., 2017; Li et al., 2023). Pediatric patients represent the most distinctive population. Compared with adults, neonates and children exhibit marked differences in PK parameters (Wacharachaisurapol et al., 2020; Salvatore et al., 2011; Yang et al., 2024). This is attributed to their immature renal function and distinct body water composition, however, body weight and CrCL remain significant factors affecting CL (Wang P.-L. et al., 2022; Antachopoulos et al., 2021). Notably, a PopPK study further revealed that SIRS also exerts a significant impact on the CL of CMS in pediatric patients (Antachopoulos et al., 2021). Importantly, the covariates identified in PopPK models should not be interpreted merely as statistical predictors. Many of them reflect specific pathophysiological processes, such as renal elimination, capillary leakage, hemodilution, extracorporeal clearance, altered protein binding, developmental maturation, or burn-induced hypermetabolism. Mechanistic interpretation of these covariates is essential for translating PopPK findings into individualized dosing strategies (Wang et al., 2026). The most critical finding across all polymyxin Pop-PK research is the profound lack of robust external model validation. External assessments reveal that many published models have poor predictive performance (Zamri et al., 2025; Chen et al., 2022; Ha et al., 2021). This deficiency is a direct result of ubiquitous limitations: small sample sizes, single-center designs, and heterogeneous reporting standards. These limitations prevent the development of universal, reliable dosing algorithms. Therefore, the TDM is strongly recommended to guide individualized dosing for these complex drugs. To propel the field forward, future research must prioritize large-scale, multi-center studies that include all polymyxins and diverse special populations. Any newly developed predictive models must undergo stringent external validation to ensure generalizability and robustness across real-world settings. Moreover, machine learning (ML)-driven approaches are crucial to unravel and model complex, underinvestigated sources of variability—that traditional methods may miss (Minichmayr et al., 2024; Hughes and Keizer, 2021). This interdisciplinary fusion of PK and ML will be essential to achieve truly personalized and optimized polymyxins (Minichmayr et al., 2024; Smith et al., 2020).

## 5 Limitations

This review is subject to limitations, primarily the difficulty in making precise cross-study comparisons. This challenge arose from fundamental differences in study designs and patient populations, compounded by the heterogeneous reporting of PK parameters (as summary statistics or model estimates). Furthermore, variability in modeling methods may have contributed to heterogeneity. Moreover, our synthesis is inevitably shaped by the original research’s constraints, frequently including single-center designs and small samples. Despite these constraints, this review successfully assembles and clarifies the current understanding of plasma PK parameters for intravenously administered polymyxins in special populations.

## 6 Conclusion

In conclusion, the profound PK variability of polymyxins, particularly in special populations, necessitates a transition from empirical dosing to more sophisticated approaches. Model-Informed Precision Dosing (MIPD), which integrates PopPK parameters, patient-specific variables, and TDM, provides a robust framework for optimizing therapy. Furthermore, the integration of ML algorithms with MIPD represents a new Frontier, holding the potential to deliver highly personalized dosing regimens that effectively balance efficacy and toxicity.
